# Stretchable piezoelectric nanocomposite generator

**DOI:** 10.1186/s40580-016-0072-z

**Published:** 2016-06-03

**Authors:** Kwi-Il Park, Chang Kyu Jeong, Na Kyung Kim, Keon Jae Lee

**Affiliations:** 1 Department of Energy Engineering, Gyeongnam National University of Science and Technology (GNTECH), 33 Dongjin-ro, Jinju-si, Gyeongsangnam-do 52725 Republic of Korea; 2 Department of Materials Science and Engineering, Korea Advanced Institute of Science and Technology (KAIST), 291 Daehak-ro, Yuseong-gu, Daejeon, 34141 Republic of Korea; 3 KAIST Institute for the NanoCentury (KINC), 291 Daehak-ro, Yuseong-gu, Daejeon, 34141 Republic of Korea

**Keywords:** Energy harvesting, Self-powered system, Piezoelectric, Stretchable nanogenerator, Flexible, Composite

## Abstract

Piezoelectric energy conversion that generate electric energy from ambient mechanical and vibrational movements is promising energy harvesting technology because it can use more accessible energy resources than other renewable natural energy. In particular, flexible and stretchable piezoelectric energy harvesters which can harvest the tiny biomechanical motions inside human body into electricity properly facilitate not only the self-powered energy system for flexible and wearable electronics but also sensitive piezoelectric sensors for motion detectors and in vivo diagnosis kits. Since the piezoelectric ZnO nanowires (NWs)-based energy harvesters (nanogenerators) were proposed in 2006, many researchers have attempted the nanogenerator by using the various fabrication process such as nanowire growth, electrospinning, and transfer techniques with piezoelectric materials including polyvinylidene fluoride (PVDF) polymer and perovskite ceramics. In 2012, the composite-based nanogenerators were developed using simple, low-cost, and scalable methods to overcome the significant issues with previously-reported energy harvester, such as insufficient output performance and size limitation. This review paper provides a brief overview of flexible and stretchable piezoelectric nanocomposite generator for realizing the self-powered energy system with development history, power performance, and applications.

## Background

Attractive approaches based on energy harvesting technology that convert ambient energy resources such as thermal, solar and mechanical energies into electrical energy have been recently studied to realize the demonstration of self-powered energy system in portable devices without external power sources like batteries [1–4]. Among these sustainable energy resources, the mechanical energy is easily accessible compared to outdoor renewable energy in anytime and anywhere (even inside human body) of our daily [5–8]. To convert the mechanical energy resources (e.g., pressure, bending, stretching and vibrational motions) to electricity, the piezoelectric energy conversion are proposed and investigated by many research groups [3, 9, 10]. In particular, the flexible energy harvester called a nanogenerator which can generate electrical energy from not only mechanical energy but also tiny biomechanical energy (e.g., heartbeat, muscle motions, and eye blinking) have attracted attention in response to the demands of infinite self-powered sources for operating flexible and wearable electronic systems [11].

**Fig. 1:**
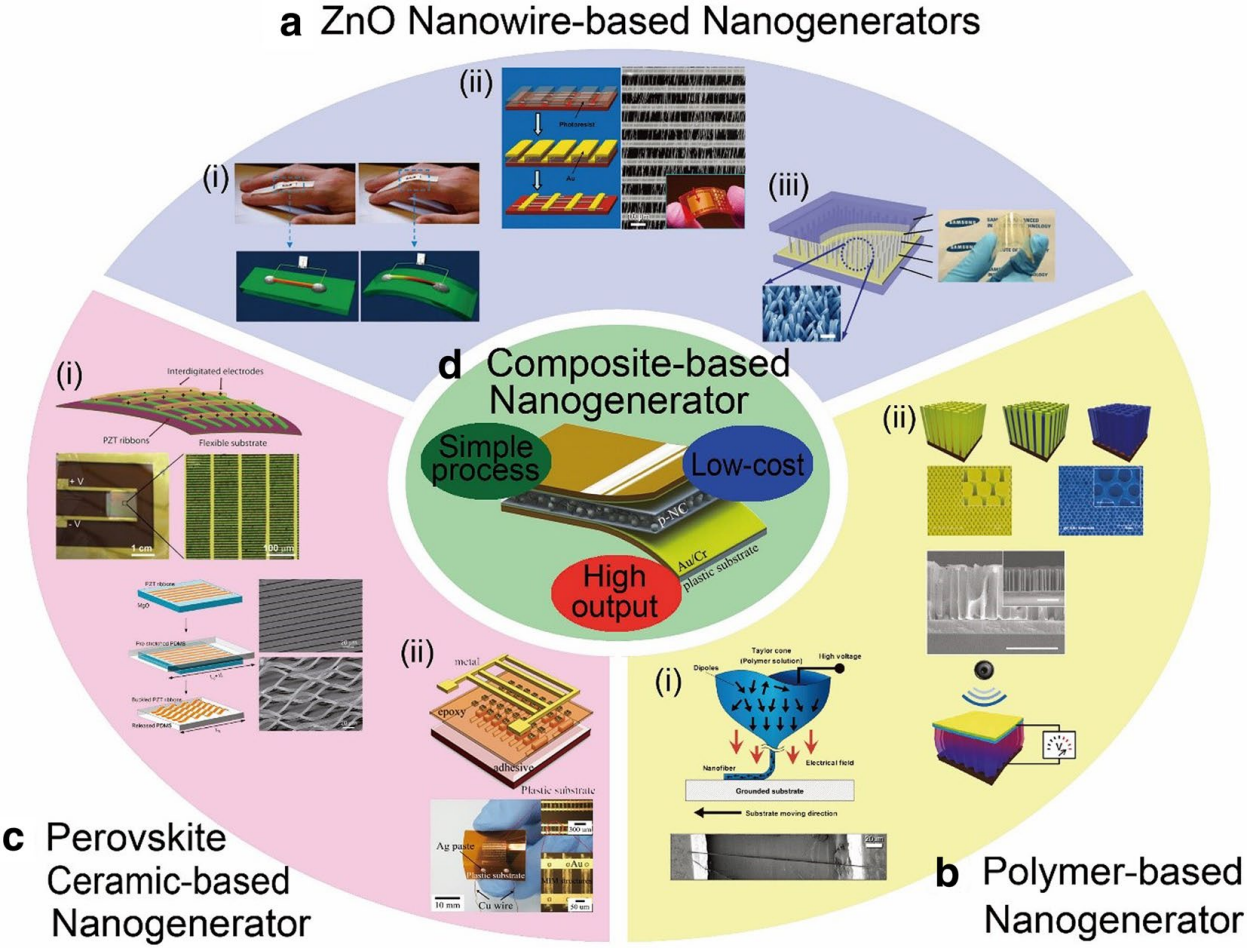
Previously reported flexible energy harvesting devices. Nanogenerators based piezoelectric materials such as (**a**) ZnO NW (reproduced from ref. [12, 14, 15] with permission, American Chemical Society and Wiley–VCH). **b** PVDF polymer (reproduced from ref. [16, 18] with permission, American Chemical Society) **c** Perovskite ceramic thin film (reproduced from ref. [19, 21, 22] with permission, American Chemical Society and Royal Society of Chemistry)

The first nanogenerator is the piezoelectric ZnO nanowires (NW) based energy device proposed by Wang and co-workers, as shown in Fig. 1a [12–15]. A single ZnO NW on a flexible polyimide (PI) substrate successfully convert from the human finger motions (Fig. 1a-i) and the running/scratching motions of a hamster to electricity [12, 13]. They also have used the transferred lateral ZnO NW arrays on a flexible substrate to enhance the output performance of nanogenerator [14], that have provided the technical advancement to operate commercial electronic devices using the nanogenerator technology (Fig. 1a-ii). Choi et al. [15] reported the transparent and flexible nanogenerator which involves the ZnO nanorod (NR) arrays and indium tin oxide (ITO) electrodes coated polyether sulfone (PES) flexible substrates (Fig. 1a-iii).

Because polymer materials have the naturally flexible properties and mechanical stabilities, the piezoelectric polymers as polyvinylidene fluoride (PVDF) have been used to fabricate polymeric flexible nanogenerators (Fig. 1b) [16–18]. Figure 1b-i presents the piezoelectric PVDF nanofibers placed on a working substrate using a unique direct-write technique. The PVDF based nanogenerator generates electrical outputs of about 5–30 mV and 0.5–3 nA when the substrate is deformed by stretching and releasing motions [16, 17]. Cha et al. [18] proposed the polymeric nanogenerator made of porous PVDF by employing a novel template-assisted method (Fig. 1b-ii). This sonic wave driven energy harvester produces higher output performance compared to dense PVDF based nanogenerators due to the effective nanoporous structure.

In 2010, there have been new approaches to use perovskite-structured ceramic [PbZr_x_Ti_1−x_O_3_ (PZT) and BaTiO_3_] thin films with inherently high piezoelectricity for higher energy conversion efficiency (Fig. 1c) [19–22]. The PZT and BaTiO_3_ thin films on bulk substrate are transferred onto flexible PI substrates by adopting unique transferring techniques after high temperature crystallization process (Fig. 1c-i, ii). The perovskite thin film-based nanogenerators show higher power density compared with other flexible piezoelectric devices with the similar device structure. Qi et al. [21] presented the energy harvester of wavy piezoelectric PZT ribbons, that can scavenge stretching motions to generate.

Recently, the research on large-area, low-cost, mechanically-stable, and high-output nanocomposite generator has been invented by using simply casting piezoelectric nanocomposites onto flexible plastic substrates at low temperature (Fig. 1d) [23–27]. In particular, several research groups have developed lead-free and high-performance flexible energy harvesters using bio-eco-compatible piezoelectric ceramics such as BaTiO_3_ [23], (K, Na)NbO_3_ [24, 25], and Li-doped (K, Na)NbO_3_ [27]. Subsequently, a new concept of ultra-stretchable elastic-composite generator, was also developed by employing the Ecoflex silicone rubber-based piezoelectric composites and long silver nanowire-based stretchable electrodes [28]. This review paper highlights a brief overview of flexible and stretchable piezoelectric nanocomposite generator for realizing the self-powered energy system, summarizing development history, power performance, and energy applications. These technologies provide novel solutions to overcome the challenging issues about nanogenerators, such as insufficient output signals and size limitation for the power sources of commercial electronics.

## Review

### BaTiO_3_ nanoparticles-based flexible nanocomposite generator

**Fig. 2:**
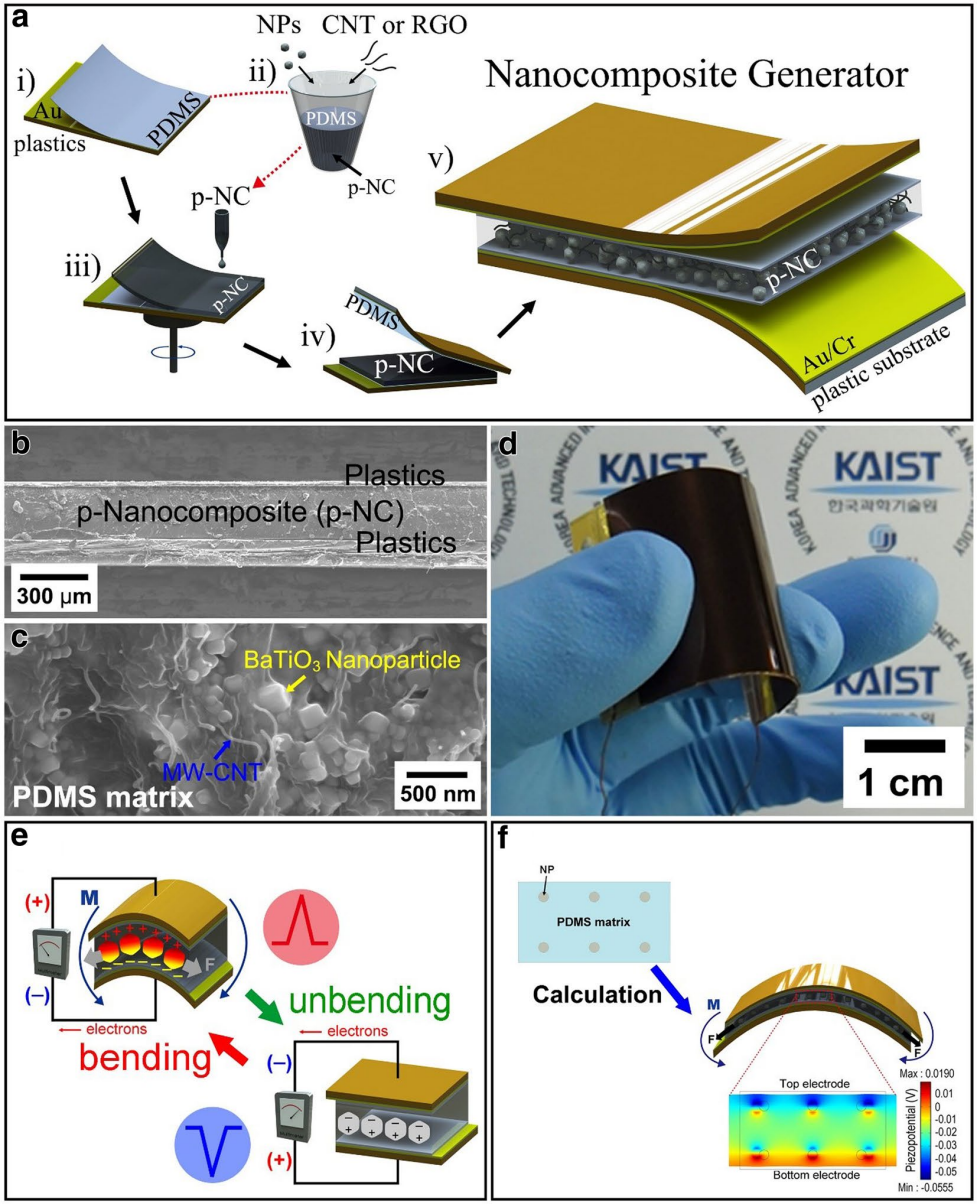
Flexible nanocomposite-based generator made of BaTiO_3_ NPs and graphitic carbons. **a** Schematic illustration showing the fabrication process of an NCG device by simple, low-cost, and scalable spin-casting. **b** A cross-sectional SEM image of an NCG device. **c** The magnified photograph of piezoelectric nanocomposite. **d** The fabricated NCG device composed of p-NC and electrode-coated plastic substrates. **e** Schematics showing power generation mechanism of the NCG device. **f** Simulation model of an NCG device consisted of six BaTiO_3_ NPs inside PDMS matrix and the calculated piezoelectric potential inside the p-NC method (reproduced from ref. [23] with permission, Wiley–VCH)

Park et al. firstly demonstrated the nanocomposite-based nanogenerator (NCG) using piezoelectric BaTiO_3_ nanoparticles and universal graphitic carbons [such as carbon nanotube (CNT) and reduced graphene oxide] by employing simple, low-cost, and large-area spin-casting/bar-coating method (Fig. 2a) [23]. A piezoelectric nanocomposite (p-NC) was produced by simply dispersing the piezoelectric BaTiO_3_ nanoparticles (NPs) and graphitic carbons within a polydimethylsiloxane (PDMS) elastomer. Note that the graphitic carbons in the NCG device play a role as dispersant avoiding precipitation of BaTiO_3_ NPs, stress agent reinforcing piezo-materials, and electrical nanobridge forming conduction paths in the polymer matrix. As shown in Fig. 2b and c, the p-NC sandwiched between electrode-coated plastic substrates has the thickness of 300 μm and the well-dispersed nanomaterials. Figure 2d presents a 3 × 3 cm^2^ sized-NCG device which can be bent by human fingers owing to the naturally flexible properties of p-NC. Figure 2e and f show the power generation mechanism of NCG device and the calculated piezopotential inside p-NC which is produced by mechanical deformation. During the regularly mechanical bending and unbending deformations, the electrons move up and down between the top and bottom electrodes; as a results, these repeat flows can generate positive and negative electric pulses (Fig. 2e). To confirm the power generation of the NCG device, the simple model consisting of six BaTiO_3_ NPs in PDMS matrix was established and calculated by multiphysics COMSOL software. Since the entire p-NC layer is tensile-stressed by bending NCG device, the piezoelectric potential is generated across the electrodes due to the piezoelectric effect of NPs (Fig. 2f). This technology can offer a significant scientific advancement for flexible piezoelectric energy harvester since it removes the demerits of previous nanogenerator such as size limitation and cost issues.

### NCG device based on various piezoelectric particles

**Fig. 3:**
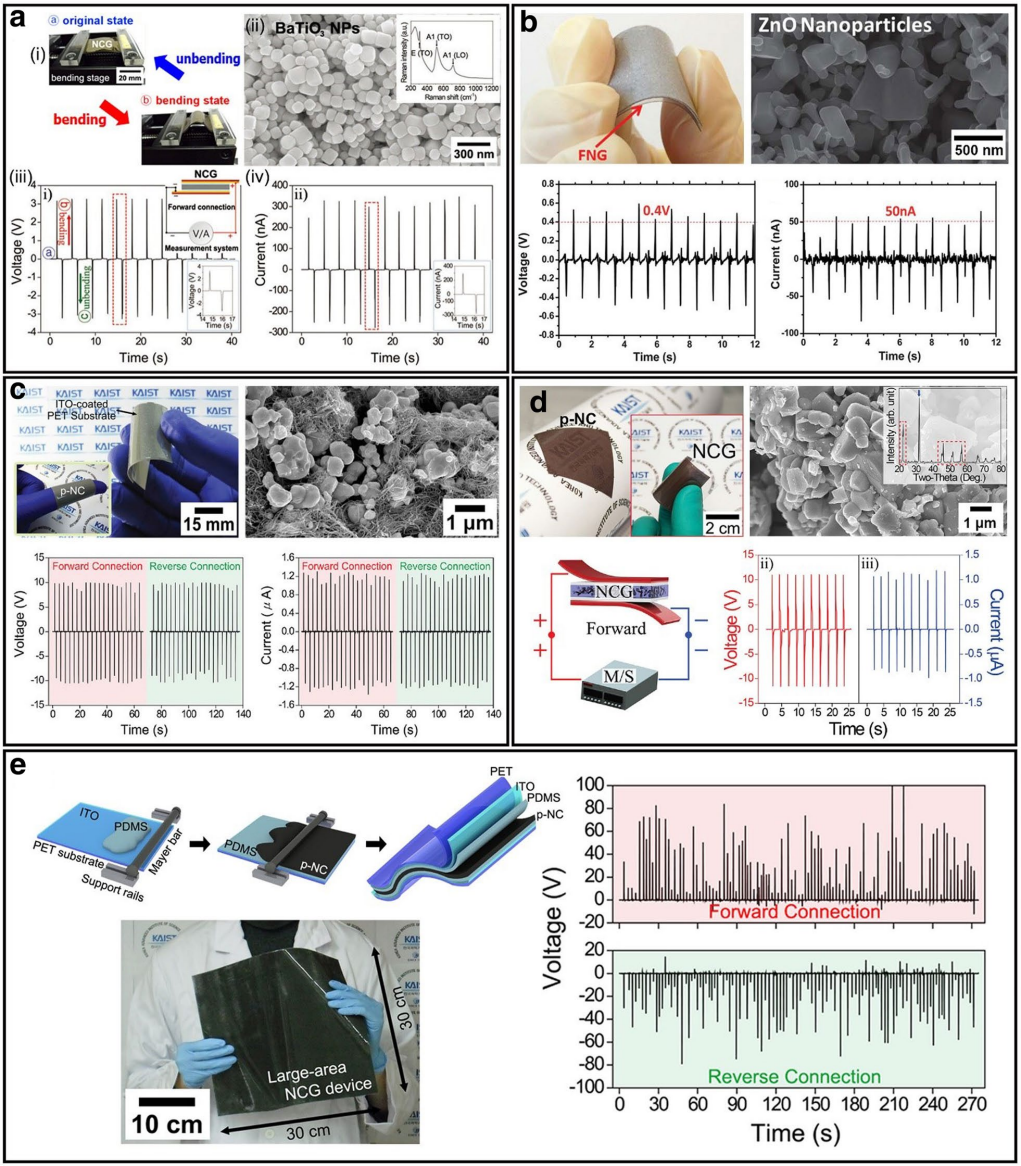
The fabricated NCG devices based on various piezoelectric particles. The generated output voltage and current signals from **a** BaTiO_3_ NPs (reproduced from ref. [23] with permission, Wiley–VCH), **b** ZnO NPs (reproduced from ref. [29] with permission, Royal Society of Chemistry), **c** PZT particles (reproduced from ref. [26] with permission, Wiley–VCH), KNLN particles (**d**) (reproduced from ref. [27] with permission, Wiley–VCH) based NCG device under periodically bending motions. **e** The large-area NCG device fabricated by a *bar*-coating technique and the measured output performance (reproduced from ref. [26] with permission, Wiley–VCH)

To characterize the output performance of NCG device, a customized bending machine of deforming the flexible devices was utilized, as shown in Fig. 3a-i. The 100 nm sized BaTiO_3_ NPs (Fig. 3a-ii)-based NCG device made an open-circuit voltage of ~3.2 V and a short-circuit current of 250–350 nA (Fig. 3a-iii, iv) during the repeatedly bending motions corresponding to displacement of 5 mm from original 4 cm long sample at strain rate of 0.2 m s^−1^ [23].

Since the first demonstration of BaTiO_3_ NPs-based NCG device, many research groups proposed other type of composite-based nanogenerators composed of various piezoelectric materials such as ZnO, PZT, and alkaline niobate particles using the NCG technique [26, 27, 29], due to the advantage of highly-efficient and large-area energy harvesters with simple and low-cost process. By using ZnO NPs of the higher piezoelectric performance compared to ZnO nanowire, the novel flexible nanogenerator composed of ZnO NPs and multiwall-carbon nanotubes (MW-CNTs) was demonstrated, as shown in Fig. 3b [29]. The energy harvester generated output voltage of 0.4 V and current pulse of 50 nA during the repeat finger gestures with the frequency of 1 Hz. The voltage and current signals reached up to the 7.5 V and 2.5 μA, respectively, under hammer knocking on the energy device. The inherently excellent piezoelectric PZT particles were also adopted to fabricate NCG device (Fig. 3c) [26]. The NCG device with PZT particles and multiwall (MW)-CNTs showed the distinguishable improvement in output performance of NCG device (3 × 3 cm^2^): the converted output voltage and current signals are ~10 V and 1.3 μA, respectively. Although this nanogenerator provided the methodology for high-output flexible energy harvesting device, PZT-based device has critical environmental drawbacks due to Pb-related problems. One of the most attractive lead-free piezoelectric materials is alkaline niobate which provides similar piezoelectric properties compared to PZT material. Jeong et al. [27] constructed the lead-free NCG device made of outstanding piezoelectric and bio-ecofriendly (K,Na)NbO_3_-LiNbO_3_ (KNLN) nanoparticles and well-dispersible copper nanorods (NRs) fillers (Fig. 3d). The KNLN-based NCG device not only creates high output with 12 V and 1.2 μA but also shows excellent stability and durability without any degeneration under the repeated bending cycles. By employing the bar-coating method, a large-area NCG device of 30 × 30 cm^2^ was demonstrated, as shown in Fig. 3e [26]. A large-scale NCG device harvested high output signals, output voltage of ~100 V and current of ~10 μA.

### Piezoelectric nanowire/tubes-based NCG device

**Fig. 4:**
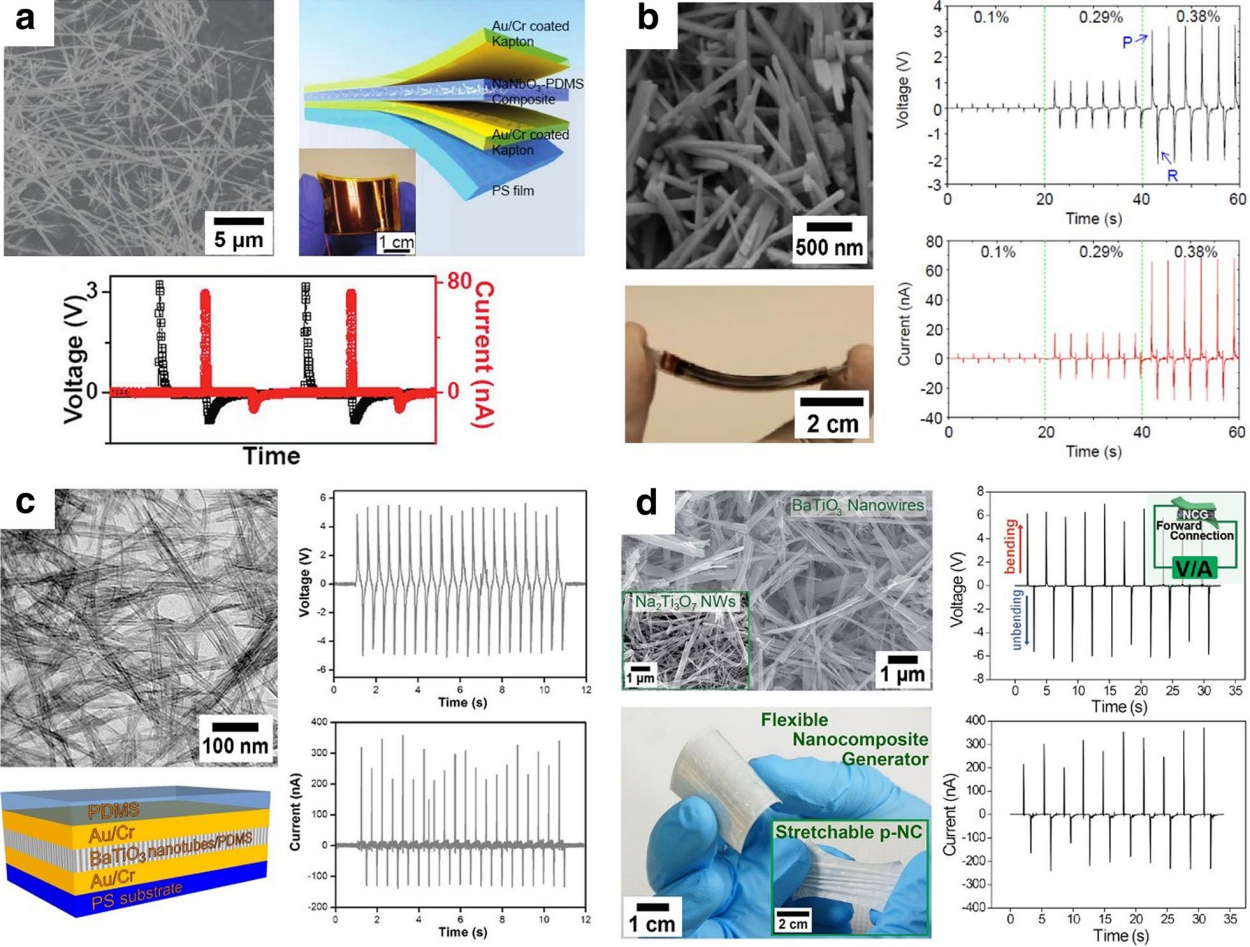
The only piezoelectric nanowires/tubes-based NCG devices. The photographs and output performance of energy harvesters made of NaNbO_3_ NWs (**a**) (reproduced from ref. [24] with permission, American Chemical Society), KNbO_3_ NRs (**b**) (reproduced from ref. [25] with permission, IOP Publishing), BaTiO_3_ NTs (**c**) (reproduced from ref. [30] with permission, American Chemical Society) and NWs (**d**) (reproduced from ref. [31] with permission, Royal Society of Chemistry)

To demonstrate the high-output NCG devices based on piezoelectric NPs, the toxic dispersant such as MW-CNTs and Cu NRs should be inevitably used to avoid the aggregation of piezoelectric NPs and enhance the output performance of electrical generation. Some research groups developed the lead-free and eco-friendly NCG devices made of non-toxic piezoelectric one-dimensional nanostructure (i.e., nanowire and nanotube) without harmful dispersing agents. Jung et al. reported the NCG device using lead-free NaNbO_3_ NWs [24] and KNbO_3_ NRs [25] as shown in Fig. 4a and b, respectively. NaNbO_3_ NWs and KNbO_3_ NRs were synthesized via hydrothermally grown method at low temperature; as a result, NaNbO_3_ NWs and KNbO_3_ NRs showed the length of ~10 μm with diameter of ~200 nm and length of ~1 μm, respectively. The lead-free alkaline-based piezoelectric materials-PDMS polymer composites were sandwiched by top and bottom electrodes-coated PI substrates and then were bent by periodically mechanical agitations. Consequently, the two NCG device converted similar output performance with open-circuit voltage of 3.2 V and ~70 nA. Figure 4c and d show the bio-eco-compatible NCG devices achieved by using the lead-free piezoelectric BaTiO_3_ nanotube [30] or nanowires [31]. Lin et al. [30] synthesized the BaTiO_3_ nanotubes by hydrothermal method and formed the p-NC by dispersing process. The flexible and transparent BaTiO_3_ nanotubes-based harvester converted the outputs of 5.5 V and 350 nA under a stress of 1 MPa (Fig. 4c). The lead-free piezoelectric BaTiO_3_ NWs synthesized by hydrothermal method at low temperature shows the average length of ~4 μm with a high aspect ratio (Fig. 4d) [31]. The BaTiO_3_ NWs could be well-distributed in PDMS elastomer without dispersing agents. Under the periodically bending and unbending motions, the output voltage and current generated from NCG device were ~7.0 V and ~360 nA, respectively. These measured values are higher than those of previous NWs-based NCGs: the high energy conversion is caused by adopting the nanostructures with high aspect ratio which can be well distributed in PDMS matrix without any dispersant agent.

### NCG device by adopting unusual structure and Bioinspired approach

**Fig. 5:**
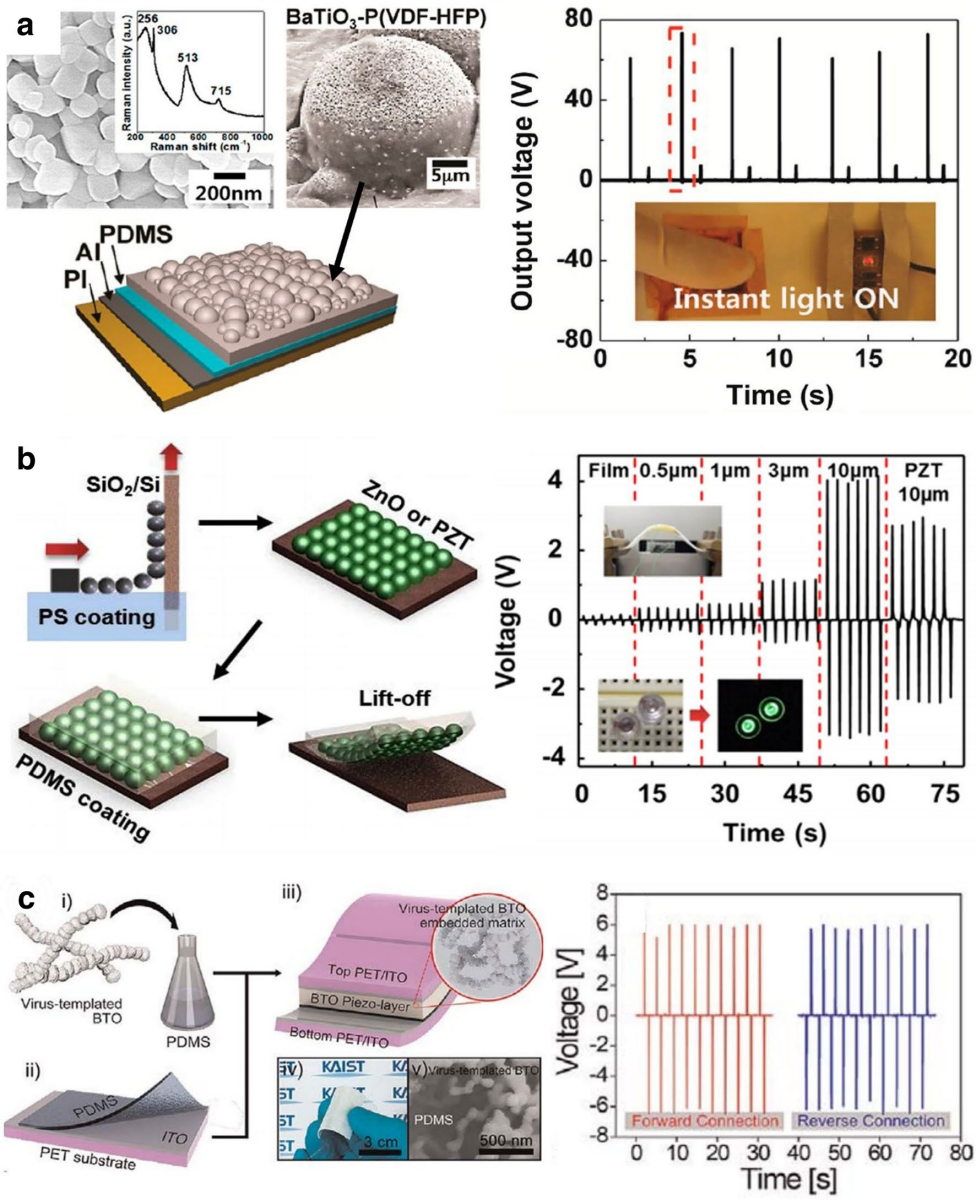
The NCG devices fabricated by new approaches. **a** The output voltage harvested from the energy harvester composed of hemispherically aggregated BaTiO_3_ NPs/P(VDF-HFP) polymer(reproduced from ref. [32] with permission, American Chemical Society). **b** The highly-stretchable composite film-based nanogenerator and its output performance (reproduced from ref. [33] with permission, Elsevier). **c** The fabrication process and output voltage of virus-templated BaTiO_3_ NWs-based NCG device (reproduced from ref. [34] with permission, American Chemical Society)

Since the key issues of energy conversion efficiency in composite-based energy harvesters depend on how to uniformly disperse the piezoelectric nanomaterials inside the matrix, it is essential to add the supplementation as filler or dispersant for enhancing the distribution. Recently, Shin et al. described high-performance piezoelectric NCG composed of hemispherically aggregated BaTiO_3_ NPs and poly-(vinylidene fluoride-co-hexafluoropropene) [P(VDF-HFP)] which formed the clusters by solvent evaporation (Fig. 5a) [32–34].The aggregated cluster formation improved the piezoelectric effect by the increment of total dipole moments inside the composite. The hemispherically aggregated BaTiO_3_ NPs composite-based NCG device demonstrated the energy generation of ~75 V and ~15 μA at applied pressure of ~0.23 MPa. Stretchable composite film-based piezoelectric energy harvester was fabricated by the piezoelectric hemispheres (Fig. 5b) [33]. The highly-ordered piezoelectric hollow hemisphere embedded composite was obtained by the deposition of ZnO or PZT thin films on a close-packed monolayer polystyrene (PS) beads template. The NCG device consisting of the 10 μm hemispheres embedded composite film produced the output voltage of ~4 V from convex bending which is approximately 8 times higher than outputs under concave motion at same strain (0.425 %). These results induced by the strong electric dipole alignment inside composite provided the feasibility of the directional anisotropic energy generation with outstanding mechanical stability. Moreover, Jeong et al. [34] demonstrated the NCG device using the unique network of anisotropic and crystalline BaTiO_3_ nanostructures which are synthesized through the biological self-assembly of multiple metal ions on genetically modified M13 viruses. This fabrication process was mostly performed in an aqueous environment under ambient conditions without toxic chemistry, suggesting an ecofriendly, energy-efficient pathway for the fabrication of a BaTiO_3_-based NCG. The bio-templated energy harvester produced the electrical outputs up to ~300 nA and ~6 V which can fully operated the LED-optical fibers and LCD devices without using any additional structural stabilizers and external sources.

### Hyper-stretchable composite energy harvester

**Fig. 6:**
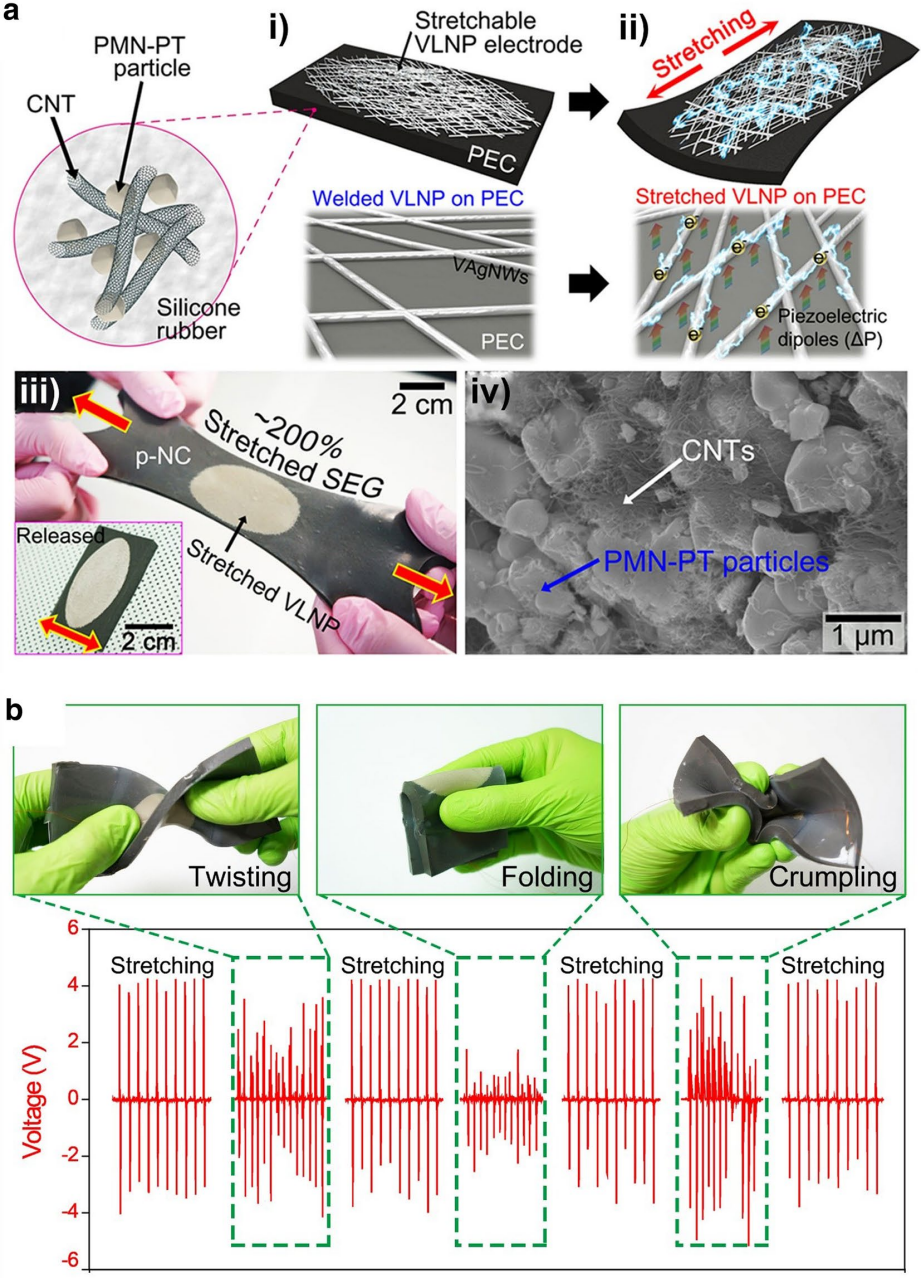
Hyper-stretchable composite energy harvester. **a** Schematic diagram of the hyper-stretchable NCG device based on PMN-PT piezoelectric composite and VAgNPN. **b** The harvested output voltage and current pulse when subjected to various deformations (twisting, folding, and crumpling) (reproduced from ref. [28] with permission, Wiley–VCH)

The stretchability of energy conversion devices also lies in a crucial need to achieve the direct and conformal integration of the stretchable electronic energy sources for various new applications such as electronic skins (e-skins), biomedical devices, and biological sensor network. Although the elastomeric composite-type nanogenerators have been considered as stretchy and freely-deformable piezoelectric energy harvesting systems, the truly reversible and stretchable NCG (strain over 20 %) has not been realized yet due to the absence of properly-conformal stretchable electrodes with large coverage and the limited elongation of general polymeric matrix. Jeong and coworkers firstly demonstrated an ultra-stretchable piezoelectric energy harvester via the the very long Ag NWs percolation network (VAgNPN) electrodes and ecoflex piezoelectric nanocomposite materials (Fig. 6a) [28]. The very long Ag NWs (VAgNWs) with average length of ~150 μm and maximum length of ~500 μm were synthesized by a novel successive multistep growth (SMG) method [35]. Using the highly-percolated VAgNWs electrodes after suction transferring onto elastomers, the stretchable electrode exhibited noteworthy conductivity (~9 Ω/sq) and remarkable stretching strain (~460 %) without electrical and mechanical failure. Ecoflex silicone rubber was chosen for the matrix of piezoelectric composite because it is hyper-stretchable elastomer up to ~900 %. The mixture of (1 − x) Pb(Mg_1/3_Nb_2/3_)O_3_—x PbTiO_3_ (PMN-PT) microparticles and MW-CNTs was well blended and dispersed in the silicone rubber. After curing the hyper-stretchable elastomeric p-NC, the VAgNWs were transferred on the p-NC by the solution filtration method. By 200 % stretching stimulations, the ultra-stretchable composite generator generated voltage of ~4 V and current of ~500 nA, respectively, which were five times higher than the poor output of the previously-reported mediocre semi-stretchable piezoelectric nanogenerators. Surprisingly, the ultra-stretchable piezoelectric generator showed the good mechanical and electrical resistance under diverse mechanical deformations such as twisting, folding (extremely bending), and crumpling (Fig. 6b). Moreover, it could directly produce the electricity signals by all kinds of mechanical stresses.

### Energy applications by flexible and stretchable NCG devices

**Fig. 7:**
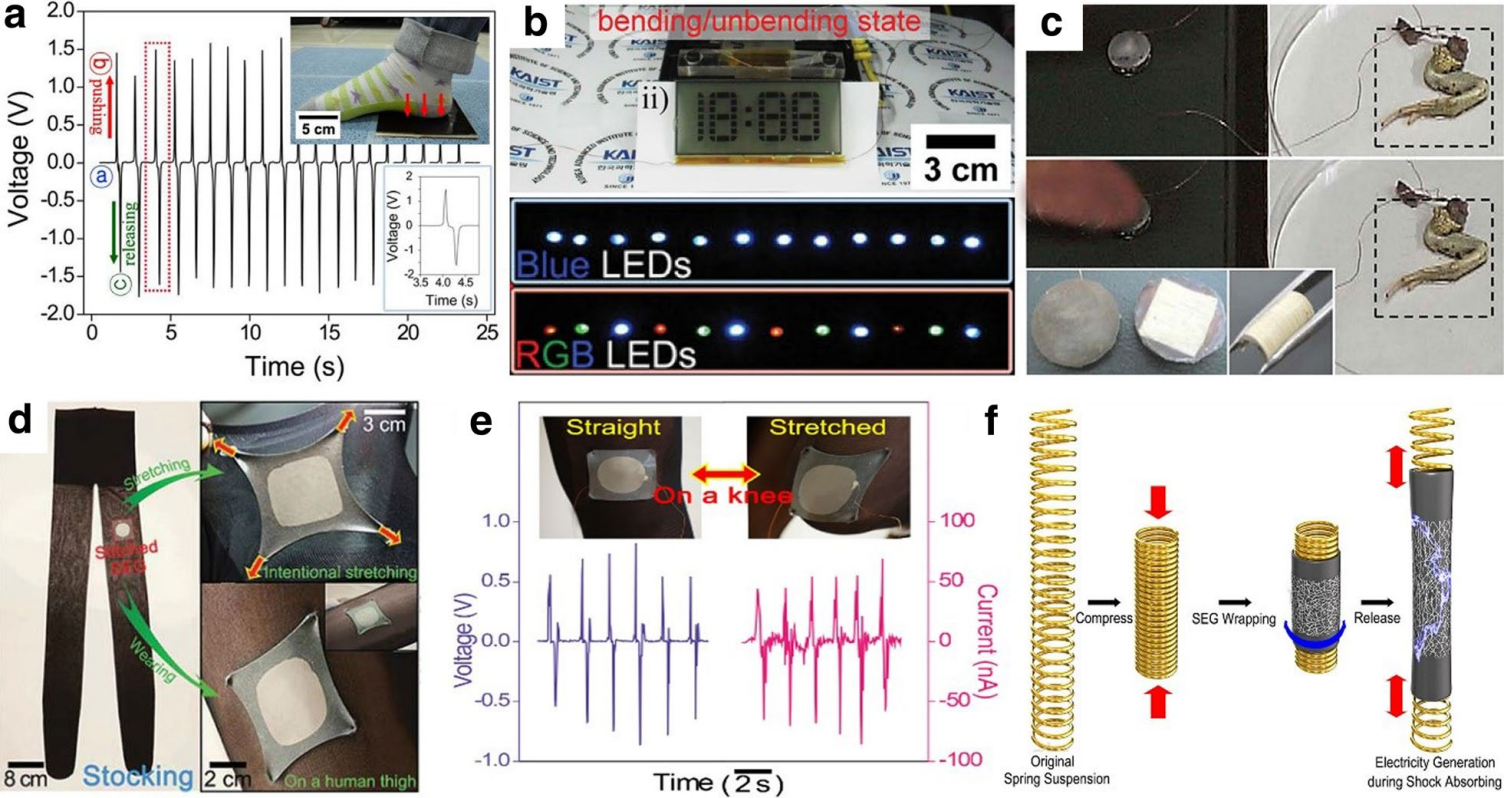
Energy applications by flexible and stretchable NCG devices. **a** The electrical energy generated from an NCG pad driven by human muscle movement (foot stepping) (reproduced from ref. [23] with permission, Wiley–VCH). **b** The commercial electronic devices such as LCD and LEDs operated by the harvested electricity (reproduced from ref. [26] with permission, Wiley–VCH). **c** The captured images showing the stimulation of a frog’s sciatic nerve by an NCG device (reproduced from ref. [36] with permission, American Chemical Society). **d** Photographs of the hyper-stretchable NCG stitched on a nylon stocking (reproduced from ref. [28] with permission, Wiley–VCH). **e** The converted electrical voltage and current from an NCG device under repeatedly stretching (reproduced from ref. [28] with permission, Wiley–VCH). **f** The schematic diagram of hyper-stretchable NCG attached on spring-based suspensions. The NCG device can produce power during shock absorbing of spring

The NCG devices can be used as not only promising energy harvesters that can generate electricity from slight movements by natural and human, but also sensitive sensors which can monitor the weak forces or motions at the wide range of physical and biological fields. Furthermore, the output voltage and currents produced from NCG devices are sufficient to operate the commercial electronic devices and stimulate the animal’s nerve. To investigate the potential utilizations of NCG technology, Park et al. [23] realized the energy harvesting that converts human muscle movement such as foot stepping into electrical energy (Fig. 7a). A BaTiO_3_ NCG pad driven by regular and slight pressure results in the repeat energy generation of ~1.5 V and ~150 nA. As shown in Fig. 7b, the commercial liquid crystal display (LCD) screen and light-emitting diodes (LEDs) were simultaneously operated by the solely electricity induced from a PZT particles-based NCG device without any external source [26]. These works showed great potential for NCGs to be commercialized in various practical electronic devices. The stimulation of NCG device to nerve is an interesting application of energy harvesting technology. Gu et al. [36] demonstrated the stimulation of frog’s sciatic nerve by ultrahigh output power (voltage of 209 V and current of 17.8 μA) from a PZT NW array-based NCG device (Fig. 7c). Moreover, the stretchable NCG device was stitched on the stretchy fabrics like a stocking for the wearable energy harvesting system, as shown in Fig. 7d. The inset pictures of Fig. 7e presents the stretched energy harvester by biomechanical kneeling and releasing motions, resulting in periodical voltage signals of ~0.7 V and current pulses of ~50 nA (Fig. 7e) [28]. This excellent electromechanical compliance of the ultra-stretchable composite nanogenerator would be imagined as energy harvesting modules in transport systems such as automobile spring-based suspensions of seats and frames, as shown in Fig. 7f.

## Conclusions

The developments of flexible and stretchable energy harvesting device through piezoelectric materials-polymer composites have enabled the self-powered energy system with high performance and improved mechanical stability. Simple and low-cost fabrication processes such as spin-casing, die-casting, and bar-coating allow the large-area energy harvester. First, the typical piezoelectric nanomaterial (e.g., BaTiO_3_ NPs) was utilized to form the piezoelectric composites; subsequently, the inherently excellent piezoelectric PZT material-based high-output NCG device were developed. Recently, the many researchers have investigated the new approaches for employing the alkaline niobate-based high piezoelectric ceramics and unusual structured BaTiO_3_ due to additional motivations for environment-friendly and biocompatible energy harvesters. Moreover, the ultra-stretchable NCG device were demonstrated via hyper-stretchable electrodes of Ag NWs percolation network in response to the demand of energy harvesting devices closely in contact with the curvy surfaces. The fabricated NCG device can produce the power from biomechanical movements and sufficiently operate the commercial electronic devices. The NCG technology will be expected to improve further by adopting the controllable lead-free piezoelectric ceramic materials [such as Ba(Zr,Ti)O_3_ (BZT), (Bi, Nd)Ti_3_O_12_, and SrBi_2_Ta_2_O_9_] and the enhaced core–shell structured nanomaterials. Furthermore, the novel printing technique with p-NC will open a key role in the development of the commercially available self-powered energy system. The piezoelectric rubbers will facilitate solutions to develop the self-powered road-system consisting of energy generation source, highly-sensitive sensor, and wireless transmitter for military and wearable applications. In addition, these unique approaches for flexible energy harvesters shall support energy sources of various state-of-a-art flexible and future electronic systems [37–56].
